# Supplementary material for: Decomposed Linear Dynamical Systems (dLDS) models reveal instantaneous, context-dependent dynamic connectivity in C. elegans
Source: Commun Biol. 2025 Aug 13;8:1218. doi: 10.1038/s42003-025-08599-3 (PMC12350842; doi:10.1038/s42003-025-08599-3)
Supplement: Supplementary file 2 — Description of Additional Supplementary Materials [file 42003_2025_8599_MOESM2_ESM.pdf]

Description of Additional Supplementary Files

File name: Supplementary Data 1

Description: Two-sided χ 2 tests for BAG and RMED activity and connectivity (inactive vs. active, defined by a threshold), behavior, and oxygen stimulation levels. Glossary: df: degrees of freedom, Q: chi squared test statistic, bhv: behavior, [neuron]connFrom: strongest connectivity value (absolute value) from that neuron, [neuron]connTo: strongest connectivity value to that neuron,[Neuron]yhat: reconstructed activity of that neuron, [ ]3: data included only above threshold 3 standard deviations above median, [ ]Pos: only positive connections included, [ ]Neg: only negative connections included, FWD: forward crawling (value= 1) vs. all other behaviors, stimTime: first half vs. second half (value= 1), stimTimesDetailed: 21% oxygen vs. 4% (value= 1).

File name: Supplementary Data 2

Description: ANOVA tests for AVFR, BAGL, BAGR, RIGL, RIS, RMED, and SABD activity and connectivity vs. behavior and oxygen stimulation factor levels. Glossary: [neuron]yhat: reconstructed activity, Bhv: behavior states (1-4), Stim: stim states (21 percent oxygen only first half trial, or alternating oxygen levels second half), df: degrees of freedom, SS: sum of squares, MS: mean squared, F: test statistic, p: p-value.

File name: Supplementary Data 3

Description: MANOVA tests for AVFR, BAGL, BAGR, RIGL, RIS, RMED, and SABD activity and connectivity vs. behavior and oxygen stimulation factor levels. Glossary: [neuron]connFrom: strongest connectivity value (absolute value) from that neuron, [neuron]connTo: strongest connectivity value to that neuron, Bhv: behavior states (1-4), Stim: stim states (21 percent oxygen only first half trial, or alternating oxygen levels second half), Bhv:Stim: Behavior: Stimulus interaction term, df: degrees of freedom, test stat: test statistic, value: value of test statistic, F: corresponding F value, dfN: degrees of freedom of numerator, dfD: degrees of freedom of denominator, p: p-value.
